# Targeted sequencing panels in Italian ALS patients support different etiologies in the ALS/FTD continuum

**DOI:** 10.1007/s00415-021-10521-w

**Published:** 2021-03-26

**Authors:** Anna Bartoletti-Stella, Veria Vacchiano, Silvia De Pasqua, Giacomo Mengozzi, Dario De Biase, Ilaria Bartolomei, Patrizia Avoni, Giovanni Rizzo, Piero Parchi, Vincenzo Donadio, Adriano Chiò, Annalisa Pession, Federico Oppi, Fabrizio Salvi, Rocco Liguori, Sabina Capellari, BoReALS

**Affiliations:** 1 IRCCS Istituto Delle Scienze Neurologiche Di Bologna, Bellaria Hospital, 40139 Bologna, Italy; 2 Dipartimento di Scienze Biomediche e Neuromotorie (DIBINEM), Università Di Bologna, 40123 Bologna, Italy; 3 Department of Pharmacy and Biotechnology, Molecular Diagnostic Unit, University of Bologna, viale Ercolani 4/2, 40138 Bologna, Italy; 4 Department of Experimental Diagnostic and Specialty Medicine (DIMES), University of Bologna, 40138 Bologna, Italy; 5 Rita Levi Montalcini Department of Neuroscience, University of Turin, Turin, Italy; 6 Azienda Ospedaliero Universitaria Citta Della Salute E Della Scienza Di Torino, Turin, Italy; 7 Neuroscience Institute of Turin, Turin, Italy

**Keywords:** Amyotrophic lateral sclerosis, Frontotemporal degeneration, Next generation sequencing, Genetic heterogeneity, Mutation screening

## Abstract

**Background:**

5–10% of amyotrophic lateral sclerosis (ALS) patients presented a positive family history (fALS). More than 30 genes have been identified in association with ALS/frontotemporal dementia (FTD) spectrum, with four major genes accounting for 60–70% of fALS. In this paper, we aimed to assess the contribution to the pathogenesis of major and rare ALS/FTD genes in ALS patients.

**Methods:**

We analyzed ALS and ALS/FTD associated genes by direct sequencing or next-generation sequencing multigene panels in ALS patients.

**Results:**

Genetic abnormalities in ALS major genes included repeated expansions of hexanucleotide in *C9orf72* gene (7.3%), mutations in *SOD1* (4.9%), *FUS* (2.1%), and *TARDBP* (2.4%), whereas variants in rare ALS/FTD genes affected 15.5% of subjects overall, most frequently involving *SQSTM1* (3.4%), and *CHMP2B* (1.9%). We found clustering of variants in ALS major genes in patients with a family history for “pure” ALS, while ALS/FTD related genes mainly occurred in patients with a family history for other neurodegenerative diseases (dementia and/or parkinsonism).

**Conclusions:**

Our data support the presence of two different genetic components underlying ALS pathogenesis, related to the presence of a family history for ALS or other neurodegenerative diseases. Thus, family history may help in optimizing the genetic screening protocol to be applied.

**Supplementary Information:**

The online version contains supplementary material available at 10.1007/s00415-021-10521-w.

## Introduction

Amyotrophic lateral sclerosis (ALS) is a devastating neurodegenerative disease characterized by the degeneration of upper and lower motor neurons. The risk of developing ALS peaks at 50–75 years of age and decreases subsequently. Survival is highly variable, but respiratory failure usually leads to death about 3–4 years after onset [1]. In up to 50% of cases, there are extra-motor manifestations such as behavior changes, executive dysfunctions, and language problems. In 10–15% of patients, these problems are severe enough to meet the clinical criteria for frontotemporal dementia (FTD) [2]. The combination of FTD with motor neuron disease (MND) refers to ALS/FTD [3]. The relationship between ALS and FTD has been confirmed by genetic studies, and these two conditions are now considered at the opposite extremes of a clinical-pathological continuum [4, 5].

Most cases are sporadic (sALS), but 10–20% of patients present a familial recurrence (fALS) [6], usually showing an autosomal dominant transmission [4]. Recent studies have highlighted the role of genetic risk factors even in sporadic patients, where heritability would represent about 21.0% [7], and twin studies have estimated a heritability of about 60% [8].

More than 30 genes have been linked to ALS and the ALS/FTD continuum [9], in addition to a small percentage of patients with sALS [10]. Four major genes, chromosome 9 open reading frame 72 (*C9orf72*)*,* superoxide dismutase 1 (*SOD1*), TAR DNA-binding protein (*TARDBP*), and fused in sarcoma (*FUS*) cover up to 60% of fALS and 10–13% of sALS cases [9]. Variants in other genes are found in < 1% of patients [10, 11].

An increasing number of cohort studies have investigated the relationship between genes and clinical phenotypes [12], as a model for researching the mechanisms underlying the disease onset and progression. They demonstrated that incomplete penetrance and genetic pleiotropy further complicate the scenario. Furthermore, more than one potentially pathogenic variant can be identified in a single patient, suggesting an oligogenic inheritance with a dose-dependent gene-burden effect [13, 14].

In this study, we compared the clinical and genetic data of Italian ALS patients to assess the genetic contribution to the pathogenesis of familial and sporadic ALS.

## Methods

### Patients

We collected clinical and genetic data from patients with definite, probable, probable laboratory-supported, and possible ALS diagnosed according to the revised El Escorial criteria [15] at the Clinica Neurologica, Bellaria Hospital (Bologna, Italy) between 2010 and 2019. Clinical data include gender, age at onset (AAO), type of onset, ALS phenotype, and family history. Regarding family history, we differentiated patients with a positive family history for ALS (fALS-ALS) from patients with a family history for other neurodegenerative diseases (fALS-ND), i.e., dementia and parkinsonism. Patients without a family history for ALS, parkinsonism or dementia were defined as sporadic (sALS).

Patients were classified into the following clinical phenotypes: classic, bulbar, predominant upper motor neuron (PUMN), and predominant lower motor neuron (PLMN) [16, 17]. Due to the relative small sample, we decided to include patients with flail arm, flail leg, and progressive muscular atrophy (PMA) variants in the PLMN group, while patients with primary lateral sclerosis were included in the PUMN category. Cognitive status was assessed through the clinical history, neurological examination, and a neuropsychological assessment including the Frontal assessment battery [18], and the Brief Mental Deterioration Battery (BMDB) [19].

For possible ALS, probable laboratory-supported ALS and probable ALS the diagnosis was confirmed during follow-up visits. The study was conducted according to the revised Declaration of Helsinki and Good Clinical Practice guidelines, and approved by the local ethics committee “Area Vasta Emilia Centro”. Informed consent was given by participants.

### Genetic analysis

#### DNA extraction

Genomic DNA (gDNA) was extracted from peripheral blood by standard procedures [13]. gDNA was quantified using the Quantus Fluorometer (Promega) with QuantiFluor double-stranded DNA system (Promega).

### Gene sequencing

All 330 patients were screened for mutations in ALS major genes [20]: *SOD1* (all exons), *FUS* (exons 6 and 15), *TARDBP* (exons 2, 3, and 5) genes and for pathogenic repeat expansion (RE) in the *C9orf72* gene as previously reported [13, 21].

Since 2015, genetic screening has been performed by next generation sequencing (NGS) multigene panels. We used one of the following custom gene panels: amplicon-based Illumina panel (TruSeq Custom Amplicon 1.5, Illumina, CA, USA) [22]; probe-based Illumina panel (Nextera Rapid Capture, Illumina); probe-based Agilent panel (Sure Select XT2, Agilent Technologies, Santa Clara, CA, USA), and Neurodegeneration Illumina panel (TruSeq Neurodegeneration, Illumina). For the purpose of this study, we analyzed only genes previously linked to ALS or ALS/FTD spectrum: *CCNF*, *CHCHD10*, *CHMP2B*, *DCTN1*, *FIG4*, *GRN*, *MAPT*, *OPTN*, *SETX*, *SQSTM1*, *TBK1*, *TREM2*, *TYROBP*, *UBQLN2*, and *VCP* (Table S1). Due to the presence of patients with a family history of dementia, we also searched for rare pathogenic variants in genes linked to other forms of dementia: *APP*, *PSEN1*, *PSEN2*, *ITM2B*, *CSF1R*, and *NOTCH3* genes (Table S1). Rare ALS genes (*ALS2*, *ANG*, *PFN1*, *SPAST*, *TUBA4A*, *UBQLN1*, and *VAPB*), and ALS risk factor genes (*NEFH*, *NEK1*, and *C21orf2*) were analyzed by Neurodegeneration Illumina panels in 51 patients (Table S1).

Enriched libraries were sequenced using MiSeq or NextSeq sequencer (Illumina), with a paired-end approach. Data were automatically analyzed by MiSeq Reporter software (Illumina) on the instrument, or by an in-house bioinformatic pipeline (Supplementary Materials). Variants were reported using the HGVS-Sequence Variant Nomenclature.

### Variant classification

Variant selection was performed with BaseSpace Variant interpreter (Illumina, CA, USA). Rare single-nucleotide variant (SNV) and small indels with a minor allele frequency (MAF) < 1% in the 1000 Genome Project database (http://browser.1000genomes.org/) or in the Genome Aggregation Database (GnomAD) [23] were selected. Variants were classified according to the American College of Medical Genetics and Genomics guidance for the interpretation of sequence variants [24]. Those reported in the ClinVar or HGMD (The Human Gene Mutation Database) databases were classified accordingly as known disease-causing variant (pathogenic) or variant of uncertain significance (VUS). Novel variants’ pathogenicity was assessed with *in-silico* effect predictor tools (Supplementary Materials).

### Statistical analysis

Statistical analysis was performed using IBM SPSS Statistics version 25 (IBM, Armonk, NY, USA). Kolmogorov–Smirnov test was used to verify the normal distribution of the data. Quantitative continuous variables were described as mean and standard deviation (SD) if the distribution was normal, or as median and range otherwise. Categorical variables were expressed as counts and percentages.

For continuous variables, the Mann–Whitney *U* or the Kruskal–Wallis tests were used to test differences between two or more groups, respectively. The chi-squared test was adopted for categorical variables and the post hoc test with Bonferroni adjustment was used if the overall chi-squared test was significant. For statistical tests, *p* < 0.05 was considered significant.

## Results

### Clinical features

A total of 330 Italian patients were included in the study and their clinical data are shown in Table 1. The median AAO was 63 years, ranging from 27 to 87 years (Table 1).

**Table 1:** Clinical features of study population

Patients/clinical characteristics	*N* (tot. 330)	%
Gender		
Male	173	52.4
Female	157	47.6
Age at onset (y)		
Median (range)	63 (27–87)	
Type of onset		
Bulbar	95	28.8
Spinal	193	58.5
Pseudo-polyneuritic	18	5.5
Pyramidal	24	7.3
ALS variant		
Classic	244	74.8
Bulbar	17	5.2
PLMN	30	10.9
PUMN	39	9.1
Deceased patients	187	56.7
Disease duration (m)		
Median (range)	35 (4–169)	
Family history		
fALS	84	25.5
fALS-ALS	30	9.1
fALS-ND	54	16.4
sALS/unknown	222	74.5
Other clinical features		
Dementia	21	6.4

*ALS* amyotrophic lateral sclerosis; *fALS* familial ALS; *fALS-ALS* familial ALS with positive family history for ALS; *fALS-ND* familial ALS with positive family history for other neurodegenerative diseases; *m* months; *N* number; *PLMN* predominant lower motor neuron; *PUMN* predominant upper motor neuron; *sALS* sporadic ALS

Overall, ALS phenotypes were distributed as follows: bulbar 5.2%, classic 74.8%, PLMN 10.9%, and PUMN 9.1%; 6.4% of all patients also showed cognitive deficits, mainly consistent with FTD. Our cohort includes 20.9% of patients with early-onset, defined as young-onset ALS (before or at the age of 50, arbitrary cut-off [25]). These patients showed a significantly higher percentage of PUMN variants than the other patients (15.9% vs. 7.3%, *p* value = 0.017).

Analysis of family history showed that 9.1% of our patients had a positive family history for ALS (fALS-ALS) (Table 1). Given the ALS/FTD continuum, we examined the percentage of patients with relatives affected by other neurodegenerative disorders, particularly dementia and/or parkinsonism (fALS-ND). These patients accounted for 16.4% of the cohort. Thus, 25.5% of our patients had a familial form of the ALS/FTD continuum [26]. Overall, fALS patients had a significantly earlier AAO (median 59 years) than sALS (median 64 years), *p* value = 0.047.

**Fig. 1:**
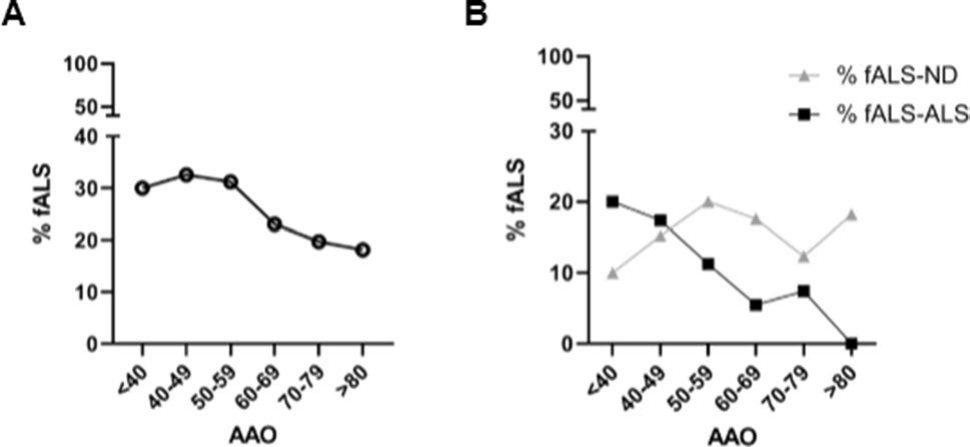
Inheritance features of the study population. **A, b** The graph shows the percentage of ALS patients with positive family history (**a**, fALS); for ALS (**b**, fALS-ALS) and for other neurodegenerative diseases (**b**, fALS-ND) stratified for age at onset (AAO)

Stratifying the familiarity for AAO, we observed a progressive decrease in familial forms after age 60 (patients with AAO before 60 had a familial form in 34.4% of cases compared to 22.3% of patients with AAO after age 60, *p* value = 0.034; Fig. 1a). However, the trend was different between fALS-ALS and fALS-ND subgroups (Fig. 1b). Indeed, the percentage of fALS-ALS was higher in patients with young-onset ALS (18.8% of ALS patients with AAO ≤ 50 as compared to 6.5% of patients with AAO > 50, *p* value = 0.007), while no differences were observed concerning the family history of fALS-ND.

Patients with cognitive decline showed a high percentage of positive family history for ALS and/or dementia/parkinsonism (55.5% vs 25.5%, *p* value = 0.004), while they did not significantly differ for AAO (*p* value = 0.558). Family history was similar among the different ALS phenotypic variants.

### Genetic screening

All patients were screened for the RE in the *C9orf72* gene and for mutations in *SOD1*, *FUS*, and *TARDBP*. 207 patients, enriched for a positive family history, were also analyzed by NGS multigene panels to verify the presence of mutations in the rare genes associated with ALS or the ALS/FTD continuum, and other dementia genes. 51 patients were also tested with a more extensive panel to test the genes reported as risk factors in ALS and other rare genes (Table S1).

### Genetic variants in the ALS major genes

Fifty-five patients (16.7%) presented a pathogenic or probably pathogenic genetic variant in one of the four major genes (Table 2). The mutation frequency was 41.7% in fALS and 8.1% in sALS. These frequencies were significantly different between fALS-ALS and fALS-ND patients (76.7% vs. 22.2%, *p* value < 0.0001).

**Table 2:** Number of pathogenic mutations in ALS-major genes identified in this study

Gene	All (*n* = 330)		fALS (*n* = 84)						sALS (*n* = 246)	
	*n*	%	*n*	%	*fALS-ALS* (*n* = 30)		*fALS-ND* (*n* = 54)		*n*	%
					*N*	%	*n*	%		
*C9orf72*	24	7.3	18	21.4	8	26.7	10	18.5	6	2.4
*SOD1*	16	4.9	8	9.5	8	26.7	0	0.00	8	3.3
*FUS*	7	2.1	4	4.8	3	10.00	1	1.9	3	1.2
*TARDBP*	8	2.4	5	6.0	4	13.3	1	1.9	3	1.2
Total	55	16.7	35	41.7	23	76.7	12	22.2	20	8.1

*ALS* amyotrophic lateral sclerosis; *fALS* familial ALS; *fALS-ALS* familial ALS with positive family history for ALS; *fALS-ND* familial ALS with positive family history for other neurodegenerative diseases; *n* number; *sALS* sporadic ALS

The pathogenic RE in the *C9orf72* gene was the most frequent mutation (*n* = 24, 7.3% of all cases, Table 2). Among these, 18 patients (21.4% of fALS) presented a positive family history, with no significant difference between the percentages in two subgroups of fALS, 26.7% among fALS-ALS cases, and 18.5% among fALS-ND cases (*p* value = 0.55). Six patients were sALS (2.4% of all sALS cases). In one patient, we identified an intermediate repeat expansion (24–30 repeats) [27].

Clinical features of *C9orf72* RE carriers are detailed in Table S2. We then identified 11 different variants in the *SOD1* gene (Tables 2 and S3), of which five classified as definite pathogenic, five as VUS, and one (c.73-4A > G), never reported, which was predicted to be likely benign (Table S4). All variants classified as VUS on HGMD or ClinVar were considered as likely causative [28–31] since for several *SOD1* variants a reduced penetrance has been described and they can, therefore, rarely be present in controls [32]. The *SOD1* variants represented the second genetic cause in our cohort (*n* = 16, 4.9% of all cases, Table 2). Eight patients were fALS, all of them with a family history of ALS (26.7% of fALS-ALS) and the remaining eight were sALS (3.3% of sALS) (Table 2).

Two novel mutations were identified in the *FUS* gene (Table S3). Both were predicted to be damaging (Tables S3 and S4). Five variants, previously described, were identified in additional five patients (Table S3). Thus, mutations in *FUS* explained 2.1% of all cases, 4.8% of fALS (10.0% fALS-ALS and 1.9% fALS-ND), and 1.2% sALS (Table 2).

Eight patients carried mutations in the *TARDBP* gene (Table 2 and S3). Variant p.Gln303His was considered likely pathogenic because it was previously described in a sALS [33]. Overall, mutations in the *TARDBP* gene were identified in 2.4% of our cohort, involving 6.0% of fALS (13.3% of fALS-ALS, and 1.9% of fALS-ND, *p* value < 0.0001), and 1.2% of sALS (Table 2).

### Genetic variants identified by NGS analysis

Among the 207 patients tested for mutations in genes rarely involved in ALS, ALS/FTD continuum, or other types of degenerative dementia (Table S1), 31 patients (14.97% of the cohort) carried pathogenic or likely pathogenic variants (Table 2). Of the 29 different variants identified, 13 were previously reported in public databases (ClinVar or HGMD) or in the literature (Table 3). Only one variant (*OPTN* p.Gln314Leu) was reported as definite pathogenic; the others were classified as VUS. In addition, we identified 16 new variants considered potentially pathogenic based on the *in-silico* prediction of pathogenicity (Tables S4 and S5).

**Table 3:** Clinical features of FTD/ALS continuum genes mutations carriers

ID patients	Gene	Variant	Classification [REF]	Gender	Family history	AAO	Variant of ALS	Additional clinical signs	Disease duration (m)	Other mutations
ALS#299	*SQSTM1*	c.86C > G p.Pro29Arg	R-VUS^a,b^	M	fALS-ND	62	Classic		> 52	
ALS#298		c.98C > T p.Ala33Val	R-VUS^a,b^ [51]	F	sALS	46	Classic		> 26	
ALS#261		c.241G > A p.Glu81Lys	R-VUS^a,b^	F	fALS-ND	78	Classic		40	
ALS#317		c.481C > T p.Arg161Trp	R-VUS^a^	M	sALS	62	Classic		> 41	
ALS#212		c.962G > A p.Arg321His	R-VUS^a,b^	M	sALS	70	Classic		54	
ALS#124		c.1175C > T p.Pro392Leu	R-VUS^a,b^	M	sALS	38	Classic		> 10	
ALS#23		c.1175C > T p.Pro392Leu	R-VUS^a,b^	M	sALS	42	Classic		22	
ALS#192	*APP*	c.2212G > A p.Val738Ile	N-VUS	F	sALS	40	PUMN		123	
ALS#159	*CCNF*	c.656 T > C p.Leu219Pro	N-likely pathogenic	M	fALS-ND	61	Classic		76	
ALS#126	*CHCHD10*	c.42-2A > C	N-likely pathogenic	F	sALS	45	Classic		> 40	*NOTCH3*
ALS#208		c.100C > T p.Pro34Ser	R-VUS^a,b^	M	sALS	73	Classic	Cognitive deficits	53	
ALS#18	*CHMP2B*	c.85A > G p.Ile29Val	R-VUS^a,b^	M	sALS	64	Classic		64	
ALS#157		c.85A > G p.Ile29Val	R-VUS^a,b^	M	fALS-ND	65	Classic		> 29	*TARDBP*
ALS#85		c.142A > C p.Lys48Gln	N-likely pathogenic	F	sALS	72	Classic		18	
ALS#225		c.557G > A p.Arg186Gln	N-likely pathogenic	F	fALS-ND	54	Classic		11	
ALS#65	*CSF1R*	c.1420G > A p.Val474Ile	R-VUS^a^	M	fALS-ND	67	PUMN		> 110	
ALS#283	*DCTN1*	c.71C > T p.Ala24Val	N-likely pathogenic	M	sALS	38	Classic		> 73	
ALS#181		c.71C > T p.Ala24Val	N-likely pathogenic	M	sALS	50	Classic		> 30	
ALS#325	*FIG4*	c.1424G > A p.Gly475Asp	N-likely pathogenic	M	fALS-ND	47	Bulbar		> 9	
ALS#101		c.434C > T p.Pro145Leu	N-likely pathogenic	F	sALS	87	Classic		24	
ALS#328	*ITM2B*	c.511A > C p.Thr171Pro	N-likely pathogenic	F	sALS	60	Classic		> 4	
ALS#141		c.751A > G p.Ile251Val	R-VUS [52]	M	fALS	45	PUMN	Optic atrophy, deafness, parkinsonism, dystonia, schizophrenia	72	*C9orf72*
ALS#126	*NOTCH3*	c.2203C > T p.Arg735Ter	N-likely pathogenic	F	sALS	45	Classic		> 40	*CHCHD10*
ALS#273		c.2618G > A p.Cys873Tyr	N-likely pathogenic	F	fALS-ND	57	Classic		> 29	
ALS#114	*OPTN*	c.235C > T p.Gln79Ter	N-likely pathogenic	F	sALS	80	Classic		24	
ALS#82		c.941A > T p.Gln314Leu	R-pathogenic^a,b^	F	sALS	42	Spinal		48	*SOD1*
ALS#230	*SETX*	c.430A > G p.Asn144Asp	N-likely pathogenic	F	sALS	50	Classic		> 55	
ALS#74		c.1255A > G p.Met419Val	N-likely pathogenic	M	sALS	72	Classic		5	
ALS#135		c.4982C > G p.Pro1661Arg	R-VUS^a^	F	sALS	51	Classic		113	*SOD1*
ALS#215	*TBK1*	c.225G > C p.Glu75Asp	N-likely pathogenic	F	sALS	61	Classic Cognitive deficits		> 37	
ALS#239		c.802A > G p.Ser268Gly	N-likely pathogenic	F	fALS-ND	65	Classic		> 19	
ALS#161	*UBQLN1*	c.162G > T p.Glu54Asp	R-VUS^a,b^	M	fALS-ND	59	Classic		> 109	

Only patients who carried pathogenic/likely pathogenic variants have been reported (pathogenic prediction of novel variants are shown in Tables S4 and S5). The clinical characteristics of patients who carried novel variants predicted as likely benign are in Table S7

*AAO* age at onset; *ALS* amyotrophic lateral sclerosis; *fALS* familial ALS, *fALS-ALS* familial ALS with positive family history for ALS; *fALS-ND* familial ALS with positive family history for other neurodegenerative diseases; *m* months; *N* novel variant; *PLMN* predominant lower motor neuron; *PUMN* predominant upper motor neuron; *R* reported variant; *REF* reference; *VUS* variant of uncertain significance

^a^Variants reported in ClinVar

^b^Variants reported in HGMD

The genes in which we identified more variants were *SQSTM1* (*n* = 7, 3.4% of the cohort) and *CHMP2B* (*n* = 4, 1.9%). We also identified variants in: *CHCHD10* (*n* = 2), *CCNF* (*n* = 1), *DCTN1* (*n* = 2), *FIG4* (*n* = 2), *OPTN* (*n* = 2), *SETX* (*n* = 3), *TBK1* (*n* = 2), and *UBQLN1* (*n* = 1).

Finally, we identified additional rare variants in non-FTD dementia related genes such as *APP, CSF1R, ITM2B,* and *NOTCH3* (Table 3). All patients carrying variants in *APP, CSF1R, ITM2B* (p.Ile251Val), and *NOTCH3* genes showed a family history for dementia.

Four patients also carried additional pathogenic variants in one of the ALS-major genes (Table 3).

### Comprehensive analysis of the ALS mutation spectrum with an extended panel

To assess genetic variants in additional ALS-related genes (*PFN1, ANG, NEFH, NEK1, TUBA4A, C21orf2*, and *SPAST*) (Table S1) a subgroup of patients (*n* = 51) was screened with an extended NGS panel. Rare potentially pathogenic variants were identified in eight patients (Table 4).

**Table 4:** Genetic variants identified in ALS risk factor genes

ID Patients	Gene (no of patients)	Variant	Classification [REF]	Gender	Family history	AAO	Variant of ALS	Additional clinical signs	Disease duration (m)	Other mutations (gene)
ALS#77	*ALS2*	c.3206G > A p.Gly1069Glu	VUS	M	fALS-ND	63	Classic		15	*C9Orf72*
ALS#86	*ALS2*	c.3128G > A p.Arg1043His	N-likely pathogenic	F	sALS	76	PUMN		36	
ALS#264	*ALS2*	c.1115C > G p.Pro372Arg	VUS	F	fALS-ND	62	Classic		119	
ALS#10	*NEK1*	c.3793C > T p.His1265Tyr	VUS	M	sALS	63	Classic		50	
ALS#72	*NEK1*	c.686A > G p.Tyr229Cys	VUS	F	sALS	73	Classic		> 23	*SPAST*
ALS#152	*NEK1*	c.1535C > T p.Ala512Val	VUS	F	sALS	41	Classic		133	
ALS#23	*SPAST*	c.1625A > G p.Asp542Gly	VUS [53]	M	sALS	38	Classic		> 10	*SQSTM1*
ALS#72	*SPAST*	c.98C > T p.Pro33Leu	VUS	F	sALS	73	Classic		> 23	*NEK1*

Only patients who carried pathogenic/likely pathogenic variants have been reported (pathogenic prediction of novel variants are shown in Tables S5)

*AAO* age at onset; *ALS* amyotrophic lateral sclerosis; *fALS* familial ALS; *fALS-ND* familial ALS with positive family history for other neurodegenerative diseases; *m* months; *N* novel variant; *PLMN* predominant lower motor neuron; *PUMN* predominant upper motor neuron; *REF* reference; *VUS* variant of uncertain significance

In particular, we found a variant in the *NEK1* gene in three patients, three heterozygous variants in the *ALS2* (*n* = 3), and two variants in the *SPAST* gene, rarely linked to ALS [34]. One patient carried variants in two rare genes (*NEK1* and *SPAST* genes), two an additional pathogenic variant in *SQSTM1,* and *C9orf72* genes (Table 4). No mutations have been identified in *PFN1, ANG, NEFH,* and *TUBA4A *genes*.*

### Genotype–phenotype correlation

The average AAO and disease duration in patients carrying variants in the four major ALS genes, and in ALS-rare genes are shown in Table S6.

*SQSTM1* was the most frequent gene carrying potentially pathogenic variants among the rare ALS genes explored (Table 3). Thus, to characterize a possible correlation with a specific phenotype, we considered this gene separately from other ALS-rare genes.

Although we did not find any significant difference between genetic groups, patients with *SQSTM1* mutations presented with a mean AAO lower and more similar to that of four major ALS genes, while patients carrying rare ALS/FTD genes mutations showed an AAO comparable to that of wild-type patients. Concerning the phenotype associated with the different variants, *TARDBP* patients showed a bulbar phenotype more frequently than wild-type patients (37.5% vs. 3.6%, *p* value = 0.0001), while *FUS* mutated patients showed a PLMN phenotype more frequently than wild-type, *C9orf72* and other rare genes mutated patients (57.1% vs. 10.8%, 4.2 and 0%, respectively, *p* value = 0.0001).

*SQSTM1* mutated patients displayed a classic phenotype without cognitive deficits at the time of diagnosis, with a family history for dementia in 28.6% of patients (Table 3).

Cognitive deficits were significantly more frequent in *C9orf72* than in wild-type patients (33.3% vs. 5%, *p* value = 0.0001). No cognitive deficits were found in patients carrying variants in other genes, with the exception of three mutated patients in *TARDPB* (c.883G > A p.Gly295Ser), *TBK1 (*c.225G > C p.Glu75Asp), and *CHCHD10* (c.100C > T p.Pro34Ser), respectively.

## Discussion

Genetics plays an important role in ALS and FTD, recognized as two diseases that form a broad neurodegenerative continuum. At least 10% and 40% of patients diagnosed with ALS and FTD are known to carry an autosomal dominant genetic mutation [35].

Given recent advances in gene therapy [36]_,_ it has become increasingly important to predict whether and which genes are involved in single ALS patients. However, ascertaining the genetic basis in patients diagnosed with ALS and/or FTD is a challenge, given the continuous discovery of new genes rarely associated with these diseases and the detection of patients with unresolved early-onset and positive family history [37, 38].

In this study, we presented the family inheritance features and the genetic landscape of an Italian cohort of adult onset ALS, providing the frequency of the four major ALS genes (*C9orf72, SOD1, FUS,* and *TARDBP*) and of others rare ones associated with ALS.

**Fig. 2:**
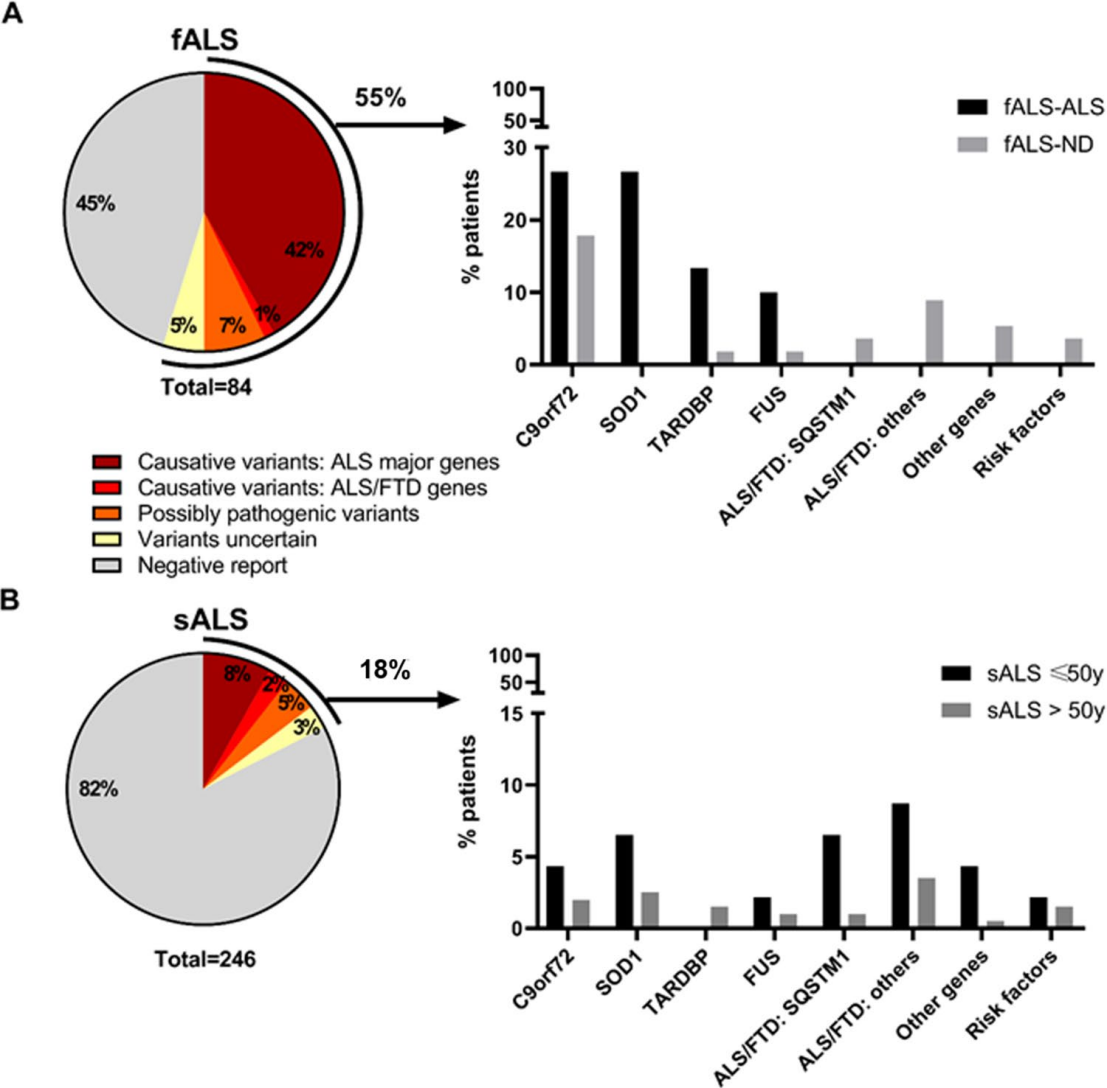
Genetic variants distribution in our ALS population. Variants are classified as causative (ALS major genes and ALS/FTD genes), possibly pathogenic variants and variant of uncertain significant (VUS). The distribution of genetic variants is different between sALS and fALS patients, accounting for 17% of sALS and 55% of fALS patients. Genetic contribution was also different between fALS-ALS and fALS-ND, and between sALS, considering AAO. *AAO* age at onset; *ALS* amyotrophic lateral sclerosis; *fALS* familial ALS; *fALS-ALS* familial ALS with positive family history for ALS; *fALS-ND* familial ALS with positive family history for other neurodegenerative diseases; *sALS* sporadic

Adding up all the molecular analyses, we identified 55 patients with a causative o possibly causative variant in the four major ALS genes, 22 with a potentially pathogenic variant in rare ALS/FTD genes (novel likely pathogenic variants or previously reported variants in ALS patients), and 9 with variants defined as of uncertain significance (novel predicted to be likely pathogenic in genes linked to other neurodegenerative diseases or in ALS risk factor genes or heterozygous variants in ALS recessive genes), (Fig. 2a,b). Of these patients, five carried more than one variant, including one with a RE in the *C9orf72* gene. In summary, 27.0% of the overall cohort carried an ALS-related variant, affecting 54.8% of fALS, and 17.5% of sALS patients (Fig. 2a,b).

Despite differences in the NGS panel and pipeline used to classify variants, these results are consistent with those reported in other high-throughput sequencing studies performed in the ALS Italian population [10, 33].

A significant proportion (45%) of fALS patients remained without a genetic diagnosis, suggesting that other ALS genes may be involved and remain to be uncovered, possibly including, as for Alzheimer’s Diseases, rare family private pathogenic variants in risk factor genes [39–41].

For the correct application of genetic testing, counseling for each patient and the interpretation of results, family history, pedigree analysis, and risk assessment remain crucial [42]. The family history for ALS in our cohort, affecting 9.1% of the cohort, was comparable to that of previous studies [43, 44]. However, when the family history for other neurodegenerative diseases was also considered, as recommended [38], the rate increased to 25.5%.

The two types of family history followed two distinct trends associated with AAO (Fig. 1b), where the family history for ALS (fALS-ALS) was associated with a lower AAO, and those with fALS-ND (family history for other neurodegenerative diseases, i.e., dementia and parkinsonism) did not differ from sALS (Fig. S1), suggesting the involvement of distinct pathogenic mechanisms and/or genetic backgrounds in these two overlapping clinical syndromes [45–47].

As for the genetic landscape, the overall mutation frequency of the four major ALS genes was 41.7% in fALS and 8.1% in sALS, similar to those observed in a recent meta-analysis [48]. These explained 76.6% of fALS-ALS cases, but only 22.2% of fALS-ND patients. The RE in *C9orf72* gene, the most frequent pathogenic mutation in our sample (Table 2) and in general in the ALS/FTD continuum in Europe [47], was associated with every phenotype of the ALS/FTD continuum and was evenly distributed in the two subgroups of fALS (Table 2).

In contrast, *SOD1* mutations were identified only in fALS-ALS (26.7% of cases), and, similarly, mutations in *FUS* and *TARDBP* were preferentially linked to fALS-ALS (Table 2).

Concerning other genes (Table S1) the most frequently involved were those belonging to the ALS/FTD continuum, rarely causative of fALS forms [11]. Their variations were detected in 32 patients (15.5% of the analyzed samples). Among these, *SQSTM1* proved to be the most frequently mutated gene, confirming previous finding in the Italian population [11] (*n* = 7, 3.4%, Table 3), followed by *CHMP2B* (*n* = 4, 1.9%, Table 3). *SQSTM1* showed a behavior similar to that of RE *C9orf72,* sharing with the major ALS genes a young AAO of the affected patients, but with a rare family history for other neurodegenerative diseases (Tables 3, S6).

Other variants were found in *CCNF*, *CHCHD10*, *DCTN1*, *FIG4*, *OPTN*, *SETX*, *TBK1*, and *UBQLN1*, and each represented less than 1% of our cohort, as previously reported [10, 11]. Together, rare ALS/FTD genes accounted for 12% of our population.

Besides, eight other patients were carriers of a likely pathogenic variant in genes not related to the ALS/FTD continuum (*APP, CSF1R, NOTCH3, ITM2B*), but linked to other types of neurodegenerative dementia. According to the model that considers the neurodegenerative disease as a continuous variation in clinical/pathological features [49], these variants may be risk factors for ALS.

Recent studies highlighted the role of genetic risk factors in sALS patients, where heritability would represent about 21.0% [7]. Likewise, we found rare variants in the *NEK1* gene, all in sALS, in 5.8% of the tested samples (3 patients out of 51) (Table 4).

Given the rarity of several mutations, we did not find any clinical features that allow us to define a specific screening pathway, as already highlighted in previous reports [42]. However, our data support the presence of at least two different pathogenetic components underlying the ALS phenotype based on the type of family history and suggest the implementation of a differentiated screening protocol.

As pointed out before, all ALS patients should be tested for RE in the *C9Orf72* gene, given the extreme variability in familial occurrence and AAO, the relatively low cost of the test and the forthcoming of *C9orf72*-targeted therapeutic trials [47]. Furthermore, fALS-ALS patients should be screened for *SOD1, FUS,* and *TARDBP,* which in our cohort explain 50% of fALS-ALS cases, reaching 76.6% with *C9orf72* testing (Fig. 2).

In contrast, patients with a family history for other neurodegenerative diseases were more likely to carry a mutation in other ALS/FTD genes (20% vs. 4.1% in fALS-ALS corresponding to only one patient, #ALS82, also carrying a *SOD1* mutation) as an inheritable trait.

In our cohort, a pathogenic/probably pathogenic mutation in the four main ALS genes or the rare ALS/FTD genes was present in 8.1% and 10% of sALS, respectively (Fig. 2). fALS may resemble a sporadic disease due to incomplete penetration or incomplete family history. In the case of negative family history, some "red flags" such as early AAO (< 50 years), atypical rapid or slow progression of the disease, and the presence of dementia may suggest a familial form [50]. No other clinical features (ALS variant, dementia, etc.) have proven useful in suggesting the possible genetic origin.

Finally, since the diagnostic algorithms should be optimized according to ethnic origin, we confirm that in the Italian population *SQSTM1* should be analyzed together with the four main genes [11].

In conclusion, despite decades of intensive research, ALS etiology remains unexplained, and the number of genes associated with disease risk and pathogenesis continues to grow. An NGS approach or exome/genome studies are not yet exhaustive and are suggested for research, hoping that they may help find a tailor-made treatment option for most ALS patients in the future. We suggest a protocol that could be useful in a clinical setting.

## Supplementary Information

Below is the link to the electronic supplementary material.

Supplementary file1 (DOCX 58 KB)

## Data Availability

The genetic data sets can be requested to the corresponding author. All clinical data are reported in the manuscript and in the supplementary data files.
