# Supplementary material for: Targeted sequencing panels in Italian ALS patients support different etiologies in the ALS/FTD continuum
Source: J Neurol. 2021 Mar 26;268(10):3766–76. doi: 10.1007/s00415-021-10521-w (PMC8463338; doi:10.1007/s00415-021-10521-w)
Supplement: Supplementary file 1 — Supplementary file1 (DOCX 58 KB) [file 415_2021_10521_MOESM1_ESM.docx]

**Supplementary Material for**

**Targeted Sequencing Panels in Italian ALS Patients support different etiologies in the ALS/FTD continuum.**

Anna Bartoletti-Stella^1^, Veria Vacchiano^1^, Silvia De Pasqua^2^, Giacomo Mengozzi^1^, Dario De Biase^3^, Ilaria Bartolomei^1^, Patrizia Avoni^1,2^, Giovanni Rizzo^1,2^, Piero Parchi^1,4^, Vincenzo Donadio^1^, Adriano Chiò^5^, Annalisa Pession^3^, Federico Oppi^1^, Fabrizio Salvi^1^, Rocco Liguori^1,2^, Sabina Capellari^1,2^on the behalf of BoReALS.

**Affiliations:**

^1^IRCCS Istituto delle Scienze Neurologiche di Bologna, Bellaria Hospital, 40139 Bologna, Italy.

^2^Dipartimento di Scienze Biomediche e Neuromotorie (DIBINEM), Università di Bologna, 40123 Bologna, Italy

^3^Department of Pharmacy and Biotechnology, Molecular Diagnostic Unit, University of Bologna, viale Ercolani 4/2, 40138 Bologna, Italy.

^4^Department of Experimental Diagnostic and Specialty Medicine (DIMES), University of Bologna, 40138 Bologna, Italy

^5^Rita Levi Montalcini Department of Neuroscience, University of Turin, Turin, Italy; Azienda Ospedaliero Universitaria Citta della Salute e della Scienza di Torino, Turin, Italy; Neuroscience Institute of Turin, Turin, Italy.

A. Bartoletti Stella and V. Vacchiano contributed equally. R. Liguori and S. Capellari share senior authorship

**Supplementary Methods**

**In-house bioinformatic pipeline for NGS analysis**

Trimming and quality assessment of raw reads was performed with fastp^54^, alignment was performed to the reference genome GRCh37/Hg19 with BWA^55^ using bwa-mem algorithm. Variant calling was performed with Strelka2^56^. For both sequencing methods, variant filtration and depth of coverage analysis were performed using Genome Analysis Toolkit (GATK)^57^ v3.8. For heterozygous variants, the allele balance value in the range of 0.30 and 0.70 was taken as threshold for further analysis. Intronic, untranslated region (UTR) and synonymous variants not predicted to affect splicing sites were removed. Variants underwent a selection based on quality criteria as well. We picked only variants whose sequence read depth was at least 10x.

**Predict the pathogenicity of novel variants**

To predict the pathogenicity of novel variants, we performed several *in silico* analyses. Functional consequences of missense variants were predicted by three *in silico* models: Polyphen2^58^, M-CAP^59^, CADD v1.4^60^, while intronic splicing variants by: NetGene2^61^, MaxEntScan^62^ and Human Splicing Finder (www.umd.be/HSF/). Variants predicted to be damaging in at least two out of three *in silico* predictor tools were defined as “likely pathogenic”.

**Supplementary Tables**

**Table S1: Genes analyzed in this study**

| **Symbol** | **Name** | **RefSeq** | **ALS role [REF]** | **Analysis method** |
| --- | --- | --- | --- | --- |
| *C9orf72* | Chromosome 9 open reading frame 72 | NM_018325.5 | ALS major gene [49] | 2-Step PCR as in Giannoccaro et al., 2017^13^ |
| *FUS* | FUS RNA binding protein | NM_004960.4 | ALS major gene [49] | Sanger or NGS Dementia panel |
| *SOD1* | Superoxide dismutase 1 | NM_000454.5 | ALS major gene [49] | Sanger or NGS Dementia panel |
| *TARDBP* | TAR DNA binding protein | NM_007375.4 | ALS major gene [49] | Sanger or NGS Dementia panel |
| *CCNF* | Cyclin F | NM_001761.3 | ALS/FTD gene [63] | NGS Dementia panel |
| *CHCHD10* | Coiled-coil-helix-coiled-coil-helix domain containing 10 | NM_213720.3 | ALS/FTD gene [10] | NGS Dementia panel |
| *CHMP2B* | Charged multivesicular body protein 2B | NM_014043.4 | ALS/FTD gene [10] | NGS Dementia panel |
| *DCTN1* | Dynactin subunit 1 | NM_004082.5 | ALS/FTD gene [10] | NGS Dementia panel |
| *FIG4* | FIG4 phosphoinositide 5-phosphatase | NM_014845.6 | ALS/FTD gene [10] | NGS Dementia panel |
| *GRN* | Granulin | NM_002087.4 | ALS/FTD gene [10] | NGS Dementia panel |
| *MAPT* | Microtubule associated protein tau | NM_005910.6 | ALS/FTD gene [10] | NGS Dementia panel |
| *OPTN* | Optineurin | NM_021980.4 | ALS/FTD gene [10] | NGS Dementia panel |
| *SETX* | Sentaxin | NM_015046.7 | ALS/FTD gene [10] | NGS Dementia panel |
| *SQSTM1* | sequestosome 1 | NM_003900.5 | ALS/FTD gene [10] | NGS Dementia panel |
| *TBK1* | TANK binding kinase 1 | NM_013254.4 | ALS/FTD gene [33] | NGS Dementia panel |
| *TREM2* | Triggering receptor expressed on myeloid cells 2 | NM_018965.4 | ALS/FTD gene [11] | NGS Dementia panel |
| *TYROBP* | TYRO protein tyrosine kinase binding protein | NM_003332.4 | ALS/FTD gene [51] | NGS Dementia panel |
| *UBQLN2* | Ubiquilin 2 | NM_013444.4 | ALS/FTD gene [10] | NGS Dementia panel |
| *VCP* | Valosin-containing protein | NM_007126.5 | ALS/FTD gene [11] | NGS Dementia panel |
| *ALS2* | Alsin Rho guanine nucleotide exchange factor ALS2 | NM_020919.4 | ALS/FTD gene [10] | NGS Neurodegeneration |
| *ANG* | Angiogenin | NM_001145.4 | ALS/FTD gene [10] | NGS Neurodegeneration |
| *PFN1* | Profilin 1 | NM_005022.4 | ALS/FTD gene [10] | NGS Neurodegeneration |
| *SPAST* | Spastin | NM_014946.4 | ALS/FTD gene [11] | NGS Neurodegeneration |
| *TUBA4A* | Tubulin alpha 4a | NM_006000.3 | ALS/FTD gene [11] | NGS Neurodegeneration |
| *UBQLN1* | Ubiquilin 1 | NM_013438.5 | MND gene [64] | NGS Neurodegeneration |
| *VAPB* | VAMP associated protein B and C | NM_004738.5 | ALS/FTD gene [10] | NGS Neurodegeneration |
| *NEFH* | Neurofilament heavy | NM_021076.4 | ALS risk factor gene [11] | NGS Neurodegeneration |
| *NEK1* | NIMA related kinase 1 | NM_012224.4 | ALS risk factor gene [11] | NGS Neurodegeneration |
| *C21orf2* | Chromosome 21 open reading frame 2 | NM_001271441 | ALS risk factor gene [63] | NGS Neurodegeneration |
| *APP* | Amyloid beta precursor protein | NM_000484.4 | Other dementia gene [51] | NGS Dementia panel |
| *CSF1R* | Colony stimulating factor 1 receptor | NM_005211.4 | Other dementia gene [51] | NGS Dementia panel |
| *ITM2B* | Integral membrane protein 2B | NM_021999.5 | Other dementia gene [51] | NGS Dementia panel |
| *NOTCH3* | Notch 3 | NM_000435.3 | Other dementia gene [51] | NGS Dementia panel |
| *PSEN1* | Presenilin 1 | NM_000021.4 | Other dementia gene [51] | NGS Dementia panel |
| *PSEN2* | Presenilin 2 | NM_000447.3 | Other dementia gene [51] | NGS Dementia panel |

Key: ALS, amyotrophic lateral sclerosis; FTD, frontotemporal dementia; ALS/FTD genes: genes known to be associated or possibly associated with ALS and/or FTD, MND, motor neuron disease; NGS, next generation sequence; RefSeq, Reference sequence; REF, reference.

**Table S2:** Clinical features of *C9orf72* RE carriers

| **ID Patients** | **Gender** | **Family History** | **Age of onset** | **ALS Variant** | **Additional clinical signs** | **Disease duration (m)** |
| --- | --- | --- | --- | --- | --- | --- |
| ALS#12 | M | sALS | 40 | PLMN |  | >13 |
| ALS#19 | M | fALS-ND | 69 | classic | cognitive deficits | >27 |
| ALS#55 | F | fALS-ND | 60 | classic |  | >11 |
| ALS#77 | F | fALS-ND | 63 | classic |  | 15 |
| ALS#79 | F | sALS | 51 | classic |  | 24 |
| ALS#81 | M | fALS-ND | 57 | classic | cognitive deficits | 29 |
| ALS#104 | F | fALS-ND | 55 | bulbar | cognitive deficits | 34 |
| ALS#106 | M | sALS | 64 | classic |  | 15 |
| ALS#116 | M | sALS | 78 | classic |  | 27 |
| ALS#141^a^ | F | fALS-ALS | 45 | PUMN | optic atrophy, deafness, parkinsonism, dystonia, schizophrenia | 72 |
| ALS#145 | F | fALS-ND | 71 | classic | cognitive deficits | >37 |
| ALS#146 | M | fALS-ALS | 34 | classic | dysautonomia | 13 |
| ALS#163 | F | fALS-ALS | 51 | classic |  | 31 |
| ALS#168 | F | sALS | 33 | classic |  | >20 |
| ALS#185 | M | fALS-ALS | 49 | classic |  | >12 |
| ALS#187 | M | fALS-ALS | 73 | classic |  | 34 |
| ALS#206 | M | fALS-ALS | 59 | bulbar | cognitive deficits | 102 |
| ALS#211 | M | fALS-ND | 58 | classic |  | >24 |
| ALS#218 | F | fALS-ND | 60 | bulbar | cognitive deficits | 15 |
| ALS#238 | F | fALS-ALS | 67 | classic |  | >34 |
| ALS#255 | M | sALS | 54 | classic |  | 37 |
| ALS#304 | F | fALS-ALS | 50 | bulbar | cognitive deficits  parkinsonism | 29 |
| ALS#312 | F | fALS-ND | 73 | classic |  | >15 |
| ALS#330 | M | fALS-ND | 41 | classic |  | >10 |

Key: ALS, amyotrophic lateral sclerosis; F, female; fALS, familial ALS; fALS-ALS, familial ALS with positive family history for ALS; fALS-ND, familial ALS with positive family history for other neurodegenerative diseases; M, male; m, months; PLMN, predomaninat lower motor neuron; PUMN, predominant upper motor neuron; sALS, sporadic ALS. ^a^previously described in Giannoccaro et al. 2017^25^.

**Table S3:** Clinical features of *SOD1*, *FUS*, and *TARDBP* mutations carriers

| **ID Patients** | **Gene** | **Variant** | **Classification^a^** | **Gender** | **Family History** | **Age of onset** | **ALS Variant** | **Additional clinical signs** | **Disease duration (m)** |
| --- | --- | --- | --- | --- | --- | --- | --- | --- | --- |
| ALS#270 | *SOD1*^b^ | c.14C>T p.Ala5Val | R-Pathogenic | M | fALS-ALS | 49 | classic |  | 22 |
| ALS#138 |  | c.14C>T p.Ala5Val | R-Pathogenic | F | fALS-ALS | 64 | classic |  | 28 |
| ALS#297 |  | c.197A>C p.Asn66Thr | R-Pathogenic^65^ | F | fALS-ALS | 74 | classic |  | 38 |
| ALS#82 |  | c.255G>C p.Leu85Phe | R-Pathogenic | M | fALS-ALS | 42 | classic |  | 48 |
| ALS#223 |  | c.272A>C p.Asp91Ala | R-VUS | F | sALS | 40 | classic |  | >98 |
| ALS#224 |  | c.272A>C p.Asp91Ala | R-VUS | M | sALS | 68 | PUMN |  | >70 |
| ALS#21 |  | c.272A>C p.Asp91Ala | R-VUS | F | sALS | 57 | PLMN |  | >115 |
| ALS#314 |  | c.272A>C p.Asp91Ala (HOM) | R-Pathogenic | M | fALS-ALS | 74 | classic | urinary urgency | 24 |
| ALS#17 |  | c.286G>A p.Ala96Thr | R-VUS | M | sALS | 64 | classic |  | >46 |
| ALS#123 |  | c.286G>A p.Ala96Thr | R-VUS | M | sALS | 44 | classic |  | >99 |
| ALS#2 |  | c.397_399delGAA p.Glu134del | RVUS | M | fALS-ALS | 47 | classic | urinary urgency | 93 |
| ALS#300 |  | c.404G>A p.Ser135Asn | R-Pathogenic | M | sALS | 79 | classic |  | 47 |
| ALS#316 |  | c.404G>A p.Ser135Asn | R-Pathogenic | M | sALS | 50 | PLMN |  | 130 |
| ALS#190 |  | c.412A>G p.Thr138Ala | R-Pathogenic | M | fALS-ALS | 59 | classic |  | >174 |
| ALS#135 |  | c.423T>A p.Ala141Ala | R-VUS | M | sALS | 51 | classic |  | 113 |
| ALS#321 |  | c.435G>C p.Leu145Phe | R-Pathogenic | F | fALS-ALS | 53 | PLMN |  | >106 |
| ALS#9 | *FUS* | c.578_621del p.Gly193_Gly207del^c^ | N-VUS | M | sALS | 74 | PLMN |  | >63 |
| ALS#232 |  | c.1542-1G>T | N-Likely pathogenic | M | sALS | 77 | classic |  | 33 |
| ALS#205 |  | c.1553G>A p.Arg518Lys | R-Pathogenic | M | fALS-ALS | 43 | classic |  | >5 |
| ALS#100 |  | c.1561C>T p.Arg521Cys | R-Pathogenic | M | fALS-ALS | 48 | PLMN |  | 26 |
| ALS#134 |  | c.1562G>A p.Arg521His | R-Pathogenic | F | fALS-ALS | 67 | PLMN |  | 45 |
| ALS#167 |  | c.1562G>A. p.Arg521His | R-Pathogenic | M | sALS | 37 | PLMN |  | 95 |
| ALS#3 |  | c.1564A>G p.Arg522Gly | R-VUS | F | fALS-ND | 37 | bulbar |  | 12 |
| ALS#97 | *TARDBP* | c.881G>T p.Gly294Val | R-Pathogenic | M | fALS-ALS | 64 | classic |  | 30 |
| ALS#229 |  | c.881G>T p.Gly294Val | R-Pathogenic | M | fALS-ALS | 52 | classic |  | >7 |
| ALS#25 |  | c.883G>A p.Gly295Ser | R-Pathogenic | M | fALS-ALS | 65 | bulbar |  | 20 |
| ALS#43 |  | c.883G>A p.Gly295Ser | R-Pathogenic | M | sALS | 53 | bulbar | cognitive deficits | >131 |
| ALS#157 |  | c.883G>A p.Gly295Ser | R-Pathogenic | F | fALS-ND | 65 | classic |  | >29 |
| ALS#83 |  | c.1144G>A p.Ala382Thr | R-Likely pathogenic | F | fALS-ALS | 36 | classic |  | 21 |
| ALS#326 |  | c.1144G>A p.Ala382Thr | R-Likely pathogenic | F | sALS | 55 | bulbar |  | >26 |
| ALS#87 |  | c.909A>C p.Gln303His | R-VUS | F | sALS | 63 | PLMN | | 31 |

Key: ALS, amyotrophic lateral sclerosis; F, female; fALS, familial ALS; fALS-ALS, familial ALS with positive family history for ALS; fALS-ND, familial ALS with positive family history for other neurodegenerative diseases; HOM, homozygous; M, male; N, novel variant; PLMN, predominant lower motor neuron; PUMN, predominant upper motor neuron; R, reported variant; sALS, sporadic ALS; VUS, Variant of uncertain significance.

The prediction of pathogenicity of novel variants is reported in Table S4 and S5, ^a^Classification reported in ClinVar (<https://www.ncbi.nlm.nih.gov/clinvar/>) or in reported study, ^b^Variants were reported using the HGVS-Sequence Variant Nomenclature, also for *SOD1,* although the conventional nomenclature assigned residue 1 to the second encoded amino acid (alanine), ^c^Deletion considered as VUS because *inframe*. Only patients who carried pathogenic/likely pathogenic variants have been reported (pathogenic prediction of novel variants are shown in Tables S4).

**Table S4**: Pathogenic prediction of splicing variants by *in-silico* tools

| **Gene** | **Variant** | **Allele frequency**  **GnomAD** | **Human Splicing finder** | **NNsplice** | **MaxEnt** | **Final classification^1^** | **ID patient** |
| --- | --- | --- | --- | --- | --- | --- | --- |
| *SOD1* | c.73-4A>G | 19/282896 | No significant impact on splicing signals | WT 0.60/MUT 0.46  (-23.33%) | WT 7.45/MUT 7.16  (-3.89%) | Likely Benign | ALS#323 |
| *FUS* | c.1542-1G>T | NR | WT 98.45/MUT70.59  (-28.3%) | WT 1/MUT 0  Site broken | WT 11.95/MUT 3.35  (-71.97%) | Likely pathogenic | ALS#232 |
| *CHCHD10* | c.42-2A>C | NR | No significant impact on splicing signals. | WT 0.57/MUT 0  Site broken | WT 9.21/MUT 1.16  (-87.40%) | Likely Pathogenic | ALS#126 |

Key: MUT, mutation. NR, not reported. WT, wild type.

^1^ Variants have been classified as “likely Pathogenic” if at least two tools out of the three used showed potentially pathogenic effects. Clinical characteristics of patients carried novel likely benign variants have been reported in Table S7

**Table S5**: Pathogenic prediction of missense variants by *in-silico* tools

| **Gene** | **Variant** | **Allele frequency**  **GnomAD** | **Polyphen2** | **M-CAP** | **CADD score** | **Final classification^1^** |
| --- | --- | --- | --- | --- | --- | --- |
| *ALS2* | c.3128G>A p.Arg1043His | 7/249526 | Probably Damaging | Possibly Pathogenic | 24.2 | Likely Pathogenic |
| *APP* | c.2212G>A p.Val738Leu | 1/109124 | Possibly Damaging | Possibly Pathogenic | 25.6 | VUS^2^ |
| *CCNF* | c.656T>C p.Leu219Pro | 10/247430 | Possibly Damaging | Possibly Pathogenic | 16.44 | Likely Pathogenic |
|  | c.697G>C p.Asp233His* | 10/244588 | Benign | Likely Benign | 18.54 | Likely Benign |
| *CHMP2B* | c.142A>C p.Lys48Gln | - | Probably Damaging | Possibly Pathogenic | 25.5 | Likely Pathogenic |
|  | c.557G>A p.Arg186Gln | 2/250258 | Benign | Possibly Pathogenic | 21.3 | Likely Pathogenic |
| *CSF1R* | c.2878G>A p.Ala960Thr* | 2/282214 | Benign | Likely Benign | 13.54 | Likely Benign |
| *DCTN1* | c.71C>T p.Ala24Val | - | Benign | Possibly Pathogenic | 21.9 | Likely Pathogenic |
| *FIG4* | c.1424G>A p.Gly475Asp | - | Possibly Damaging | Possibly Pathogenic | 23.6 | Likely Pathogenic |
|  | c.434C>T p.Pro145Leu | - | Probably Damaging | Likely Benign | 30 | Likely Pathogenic |
| *ITM2B* | c.511A>C p.Thr171Pro | 1/251376 | Probably Damaging | Possibly Pathogenic | 27.6 | Likely Pathogenic |
| *NOTCH3* | c.2203C>T p.Arg735Ter | 2/245670 | NA | NA | 34 | Likely Pathogenic |
|  | c.2618G>A p.Cys873Tyr | - | Probably Damaging | Possibly Pathogenic | 27.9 | Likely Pathogenic |
| *OPTN* | c.235C>T p.Gln79Ter | 3/251268 | NA | NA | 39 | Likely Pathogenic |
| *PSEN1* | c.130C>T p.Leu44Phe | - | Benign | Possibly Pathogenic | 7.22 | Likely Benign |
| *SETX* | c.430A>G p.Asn144Asp | - | Probably Damaging | Possibly Pathogenic | 25.1 | Likely Pathogenic |
|  | c.1255A>G p.Met419Val | 5/282584 | Probably Damaging | Possibly Pathogenic | 24.8 | Likely Pathogenic |
| *TBK1* | c.225G>C p.Glu75Asp | - | Probably Damaging | Possibly Pathogenic | 7.9 | Likely Pathogenic |
|  | c.802A>G p.Ser268Gly | 4/249330 | Benign | Possibly Pathogenic | 23.3 | Likely Pathogenic |

Key: ^1^ Variants have been classified as “likely Pathogenic” if at least two tools out of the three used showed potentially pathogenic effects. ^2^ Variant classified as VUS because not located in the exon 16 and 17 [66]. *Clinical characteristics of patients with likely benign variants have been reported in the Table S7. NA, not available.

**Table S6:** Mean onset age and mean disease duration of ALS patients carrying *C9Orf72, SOD1, FUS, TARDBP, SQSTM1* and rare ALS/FTD genes mutations.

| **Gene** | **Mean Onset Age**  **(y)** | **Std. Dev** | **Mean Disease duration (m)** | **Std. Dev** |
| --- | --- | --- | --- | --- |
| Wild-type | 59.94 | 11.964 | 44.67 | 37.434 |
| C9orf72 | 56.46 | 12.311 | 34.07 | 24.509 |
| SOD1 | 56.18 | 12.526 | 60.33 | 40.863 |
| FUS | 54.71 | 17.462 | 42.20 | 31.839 |
| TARDBP | 56.63 | 9.985 | 25.50 | 5.802 |
| SQSTM1 | 56.86 | 15.093 | 38.67 | 16.042 |
| Rare Genes | 60.52 | 12.801 | 44.22 | 38.457 |

Key: m, months. Std. Dev, standard deviation. y, years.

**Table S7:** Clinical characteristics of patients carried novel likely benign variants

| **ID Patients** | **Gene** | **Variant** | **Gender** | **Family History** | **AAO** | **Variant of ALS** | **Additional clinical signs** | **Disease duration (m)** | **Other mutations** |
| --- | --- | --- | --- | --- | --- | --- | --- | --- | --- |
| ALS#323 | *SOD1* | c.73-4A>G | M | fALS-ND | 40 | PLMN |  | > 65 |  |
| ALS#184 | *CCNF* | c.697G>C p.Asp233His | M | sALS | 65 | classic | schizophrenia | > 57 |  |
| ALS#320 | *CSF1R* | c.2878G>A p.Ala960Thr | F | sALS | 50 | classic |  | 11 |  |
| ALS#110 | *PSEN1* | c.130C>T p.Leu44Phe | F | fALS-ND | 69 | classic |  | > 21 |  |

Key: F, female; M, male; m, months; PLMN: predominant lower motor neuron.

**Supplementary Figure S1**

**
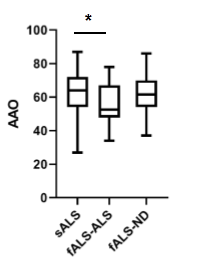
**

**Figure S1.** Age at onset of ALS familial and sporadic patients

The box plot shows the distribution of age at onset for sALS, fALS-ALS, and fALS-ND. The middle horizontal line represents the median age at onset, the upper and lower border of the box represent the value of the first quartile (25%) and the third quartile (75%) of the data, the whiskers represent the minimal and maximal AAO. A significant difference (*p<0.05) in AAO was observed between sALS and fALS-ALS.

Key: age at onset, AAO; ALS, amyotrophic lateral sclerosis; fALS, familial ALS; fALS-ALS, familial ALS with positive family history for ALS; fALS-ND, familial ALS with positive family history for other neurodegenerative diseases.

**Supplementary References**

54. Chen S, Zhou Y, Chen Y, Gu J. fastp: an ultra-fast all-in-one FASTQ preprocessor. *Bioinformatics*. 2018;34(17):i884-i890.

55. Li H, Durbin R. Fast and accurate long-read alignment with Burrows-Wheeler transform. *Bioinformatics*. 2010;26(5):589-595.

56. Kim S, Scheffler K, Halpern AL, et al. Strelka2: fast and accurate calling of germline and somatic variants. *Nat Methods*. 2018;15(8):591-594.

57. Van derAuwera GA, Carneiro MO, Hartl C, et al. From FastQ data to high confidence variant calls: the Genome Analysis Toolkit best practices pipeline. *CurrProtoc Bioinformatics*. 2013;43(1110):11.10.1-11.10.33.

58. Adzhubei I, Jordan DM, Sunyaev SR. Predicting functional effect of human missense mutations using PolyPhen-2. *CurrProtoc Hum Genet*. 2013;Chapter 7:Unit7.20.

59. Jagadeesh KA, Wenger AM, Berger MJ, et al. M-CAP eliminates a majority of variants of uncertain significance in clinical exomes at high sensitivity. *Nat Genet*. 2016;48(12):1581-1586.

60. Rentzsch P, Witten D, Cooper GM, Shendure J, Kircher M. CADD: predicting the deleteriousness of variants throughout the human genome. *Nucleic Acids Res*. 2019;47(D1):D886-D894

61. Hebsgaard SM, Korning PG, Tolstrup N, Engelbrecht J, Rouzé P, Brunak S. Splice site prediction in Arabidopsis thaliana pre-mRNA by combining local and global sequence information. *Nucleic Acids Res*. 1996;24(17):3439-3452.

62. Yeo G, Burge CB. Maximum entropy modeling of short sequence motifs with applications to RNA splicing signals. *J Comput Biol*. 2004;11(2-3):377-394.

63. Chia R, Chiò A, Traynor BJ. Novel genes associated with amyotrophic lateral sclerosis: diagnostic and clinical implications. Lancet Neurol. 2018;17(1):94-102

64. González-Pérez P, Lu Y, Chian RJ, et al. Association of UBQLN1 mutation with Brown-Vialetto-Van Laere syndrome but not typical ALS. NeurobiolDis. 2012;48(3):391-398.

65. Martinelli I, Zucchi E, Gessani A, et al. A novel p.N66T mutation in exon 3 of the SOD1 gene: report of two families of ALS patients with early cognitive impairment. Amyotroph Lateral Scler Frontotemporal Degener. 2020;21(3-4):296-300.

66. Cacace R, Sleegers K, Van Broeckhoven C. Molecular genetics of early-onset Alzheimer's disease revisited. Alzheimers Dement. 2016 Jun;12(6):733-48. doi: 10.1016/j.jalz.2016.01.012. Epub 2016 Mar 24.
